# An evaluation of large group cognitive behaviour therapy with mindfulness (CBTm) classes

**DOI:** 10.1186/s12888-019-2124-5

**Published:** 2019-05-03

**Authors:** Vishal K. Thakur, Jacquelyne Y. Wong, Jason R. Randall, James M. Bolton, Sagar V. Parikh, Natalie Mota, Debbie Whitney, Joshua Palay, Jolene Kinley, Simran Diocee, Tanya Sala, Jitender Sareen

**Affiliations:** 1 Max Rady College of Medicine, University of Manitoba, Winnipeg, Manitoba Canada; 2 Department of Psychiatry, University of Manitoba, PZ-430, 771 Bannatyne Avenue, Winnipeg, MB R3E 3N4 Canada; 3 Injury Prevention Centre, School of Public Health, University of Alberta, Edmonton, Alberta Canada; 4 Department of Community Health Sciences, University of Manitoba, PZ-430, 771 Bannatyne Avenue, Winnipeg, MB R3E 3N4 Canada; 5 Department of Psychiatry, University of Michigan, Ann Arbor, MI USA; 6 Department of Clinical Health Psychology, University of Manitoba, Winnipeg, Manitoba Canada

**Keywords:** Cognitive therapy, Large group classes, Anxiety, Depression, Psychoeducation, Mindfulness

## Abstract

**Background:**

Ensuring equitable and timely access to Cognitive Behaviour Therapy (CBT) is challenging within Canada’s service delivery model. The current study aims to determine acceptability and effectiveness of 4-session, large, Cognitive Behaviour Therapy with Mindfulness (CBTm) classes.

**Methods:**

A retrospective chart review of adult outpatients (*n* = 523) who attended CBTm classes from 2015 to 2016. Classes were administered in a tertiary mental health clinic in Winnipeg, Canada and averaged 24 clients per session. Primary outcomes were (a) acceptability of the classes and retention rates and (b) changes in anxiety and depressive symptoms using Generalized Anxiety Disorder 7-item (GAD-7) and Patient Health Questionnaire 9-item (PHQ-9) scales.

**Results:**

Clients found classes useful and > 90% expressed a desire to attend future sessions. The dropout rate was 37.5%. A mixed-effects linear regression demonstrated classes improved anxiety symptoms (GAD-7 score change per class = − 0.52 [95%CI, − 0.74 to − 0.30], *P* < 0.001) and depressive symptoms (PHQ-9 score change per class = − 0.65 [95%CI, − 0.89 to − 0.40], *P* < 0.001). Secondary analysis found reduction in scores between baseline and follow-up to be 2.40 and 1.98 for the GAD-7 and PHQ-9, respectively. Effect sizes were small for all analyses.

**Conclusions:**

This study offers preliminary evidence suggesting CBTm classes are an acceptable strategy to facilitate access and to engage and maintain clients’ interest in pursuing CBT. Clients attending CBTm classes experienced improvements in anxiety and depressive symptoms. Symptom improvement was not clinically significant. Study limitations, such as a lack of control group, should be addressed in future research.

## Background

Each year, it is estimated up to 3.5 million Canadians will access health services for a primary mood or anxiety disorder [1], and individuals with an anxiety disorder are known to be at an increased risk of developing a comorbid major depressive disorder [2]. These mental health conditions are associated with general medical conditions [3, 4], poor psychosocial functioning [5, 6], and poor occupational functioning [7, 8], leading to significant burden on both affected individuals and society [9]. Canadian clinical practice guidelines list Cognitive Behaviour Therapy (CBT) as a first line treatment for both anxiety and major depressive disorders [2, 10]. CBT is an empirically based psychotherapy with robust evidence for the treatment of adult anxiety and depression [11–13]. CBT is based on identifying and shifting clients’ dysfunctional cognitions and behaviours to reduce maladaptive emotions [14].

CBT is administered in diverse settings by a variety of health care practitioners including general practice physicians, psychiatrists, psychologists, nurses, and occupational therapists. Practitioners traditionally administer CBT to clients individually or in small group sessions, but ensuring equitable and timely access to CBT skills is challenging within this delivery model [15]. Poor access to treatment is a major issue precluding effective public health initiatives in anxiety and depression management, with a substantial proportion of individuals not receiving treatment despite a perceived need [16, 17]. Offering brief, low-intensity CBT within a stepped care model is one strategy aimed at improving CBT access in Canada [18]. Examples include self-help books, website based therapies, and, of particular interest to our study, large psychoeducational groups [19]. Administering CBT in a large-group is a promising solution which enables clinicians to reach a large number of clients.

Large-group CBT was introduced at a tertiary care clinic in Winnipeg, Canada in 2014 to manage the problem of persistently long wait times. These transdiagnostic 2-session CBT classes were rated useful by clients, led to modest improvements in anxiety symptoms, and reduced wait-times from approximately one year to three months [20]. Given these promising findings and client feedback, the CBT classes were expanded to 4 sessions and introduced mindfulness within the core content. These 4 session transdiagnostic Cognitive Behaviour Therapy with Mindfulness (CBTm) classes were independently developed and administered at the clinic to introduce clients to CBT principles, basic mindfulness strategies, and to provide various self-help resources at a time where they otherwise may not have had access to therapy.

Mindfulness is the process of being nonjudgmentally aware of the present moment, including one’s thoughts, sensations and environment, while encouraging inquisitiveness, open observation, and acceptance [21, 22].Evidence suggests mindfulness-based interventions, such as mindfulness-based cognitive therapy (MBCT) and mindfulness-based stress reduction (MBSR), are effective in treating anxiety and depression [22, 23]. Mindfulness, as it is taught in these interventions and in the CBTm classes, is intended to reduce identification with thoughts and feelings by cultivating an awareness of the impermanence (arising, passing and changing) of these mind productions. There is accumulating evidence that mindfulness meditation, with the goal of calm attentiveness and acceptance, down-regulates mental activity within the default mode network (DMN). DMN activation is associated with mind wandering, negative affect and rumination as experienced by those with anxiety or depression [24]. Other work shows that mindfulness may improve cognitive flexibility, working memory capacity, goal directed behaviour, and emotional regulation, as one’s attention and cognitive resources are shifted away from dysfunctional thoughts and emotions [25]. Moreover, these complex functions may be modulated by neural networks, whose resources can be constrained by negative emotions and mind wandering; meditation allows for a more flexible allocation of these limited resources, such that they may be available for other, more salutary, cortical functions [24].

To our knowledge, there is no research on brief, low intensity, large group CBT interventions which incorporate mindfulness in the literature. Thus, the current study sought to evaluate the 4-session CBTm class intervention in a Canadian population. We conducted a retrospective chart review of clients who attended classes between 2015 and 2016. The two primary outcomes were: (a) acceptability and retention rates of CBTm classes and (b) clients’ change in anxiety and depressive symptoms as a result of attending CBTm classes. Recent UK studies demonstrated similar large-group CBT interventions are efficient, well tolerated, and effective in treating symptoms of anxiety and depression [26–28]. Thus, we hypothesized the CBTm classes would replicate these findings by being acceptable, both in terms of client feedback and retention rates, and lead to improvements in anxiety and depressive symptoms.

## Methods

### Participants

**Fig. 1:**
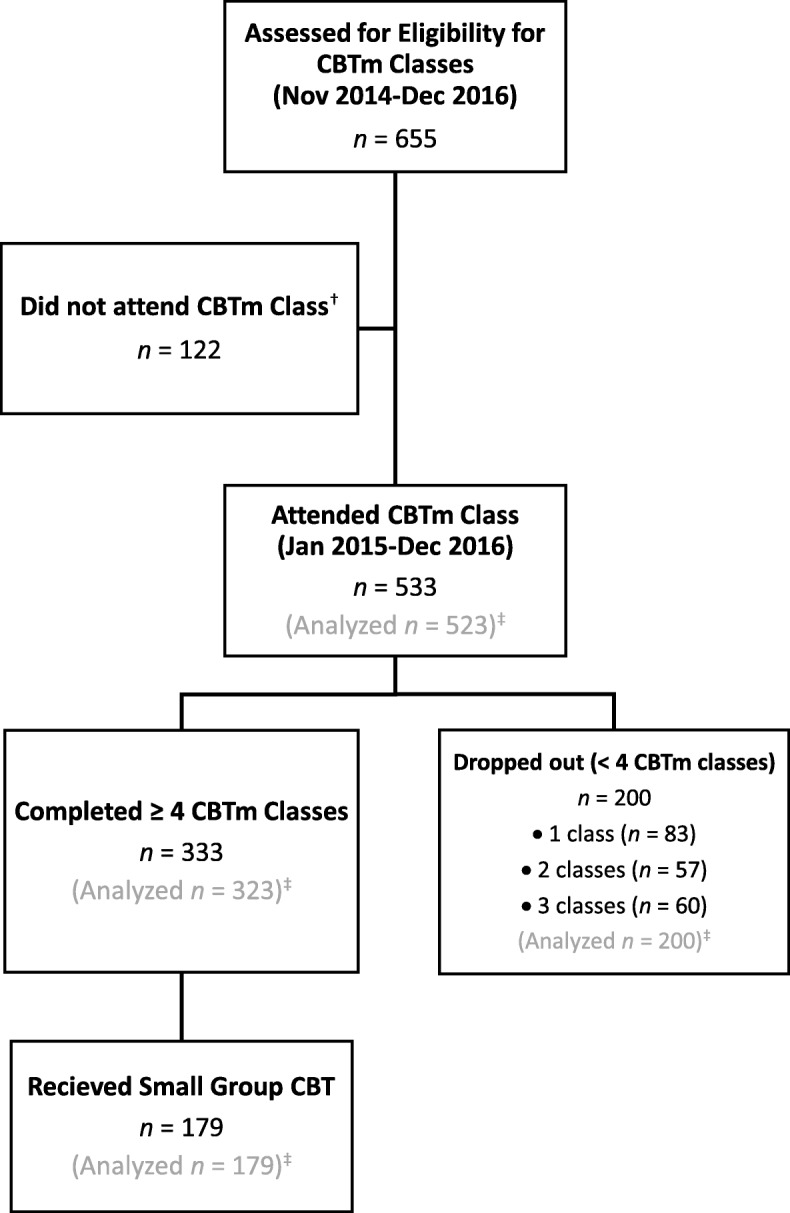
Flow of Participants Through Outpatient Mental Health Clinic. ^†^Reasons for participants not attending include meeting exclusion criteria or personal reasons for not attending. ^‡^Participants included in data analysis had to complete at least one measure in one CBTm class from Jan 2015-Dec 2016. CBT, Cognitive Behaviour Therapy; CBTm, Cognitive Behaviour Therapy with Mindfulness

Clients who attended at least one CBTm class between January 2015 and December 2016. Clients were referred by general practice physicians to a centralized intake service within a tertiary care hospital in Winnipeg, Canada. From there, clients were referred to an outpatient mental health clinic for further assessment by a psychiatrist, psychiatry resident, or nurse therapist (referrals began in November 2014). A mental health diagnosis was either confirmed or established during this interview by the clinician, although no standardized diagnostic tools were used. The presence of a mental health diagnosis and being ≥18 years of age were the only specific inclusion criteria required to be eligible for the classes. Exclusion criteria for the classes include being < 18 years of age, the presence of active psychosis or mania, acutely elevated suicide risk, or severe cognitive impairment. It was deemed these factors would potentially prevent an individual from adequately concentrating and absorbing the class content. Alternative treatments were offered to ineligible clients as clinically indicated Fig. 1.

### Intervention

Classes were 90 min in length and ranged in size from 10 to 41 clients (*M* = 24, *SD* = 7) per session, not including clients’ partners, family members, or friends who were encouraged to attend. Sessions were led and facilitated by two staff psychiatrists. One facilitator received certification from the Academy of Cognitive Therapy in 2004 and received the Beck Scholar Award in 2013. The other facilitator completed a 5-day in-person training course on Core Concepts in CBT at the Beck Institute in 2009, as well as 8 h of online training on Integrating CBT and Mindfulness through the Beck Institute’s online training program. Neither facilitator received specific training in MBSR or MBCT. Occasionally other facilitators, such as medical students and residents, assisted in running the classes. There was minimal interaction between facilitators and clients, with interactions often limited to discussions of homework and answering clients’ questions or concerns. In terms of homework, clients were encouraged to practice mindfulness meditation for five minutes, twice per day, and to partake in at least two other activities brought up in class. These homework activities included, but were not limited to, accessing self-help websites, setting goals, thought records, mood tracking, and physical exercise such as attending a yoga class. Unlike formal CBT programs, there were no individually tailored activities or specific feedback given to clients as they were progressing through homework activities and applying CBT skills. Class content was structured as follows:

- Class 1: introduction and outline of the course, rules and expectations, self-help resources, mindfulness exercise, introduction to the cognitive behavioural framework, cognitive distortions, thought records, and homework.
- Class 2: mindfulness exercise, review of homework, basics of behaviour therapy, exposure therapy, goal setting, and homework.
- Class 3: mindfulness exercise, review of homework, discussion of healthy living, sleep hygiene, and homework.
- Class 4: mindfulness exercise, review of homework, anger management strategies, assertiveness training, self-compassion, problem solving, and homework.

The four mindfulness meditations used in the CBTm classes were derived from those taught within mindfulness-based stress reduction (MBSR) [29] which is a transdiagnostic intervention proven useful with stress, depression, and anxiety management [30–32]. Specifically, the meditations were body scan, awareness of the breath, awareness of the five senses and loving-kindness. These were introduced in the same order as followed within MBSR. Clients were encouraged to download the no-cost app, MindShift, which has recorded instructions for both body scan and awareness of breath. They could also seek out recorded instructions for the other meditations but no specific direction was given for this.

Following completion of 4 classes, clients were welcome to repeat classes as booster sessions or proceed to conventional CBT group therapy if more intensive treatment was required. Verbal or written consent from each client was not required to perform the chart review. A corresponding website (cbtm.ca) was also developed in which class materials and handouts could be accessed for free.

### Procedure

The sample was derived from a retrospective chart review with approval from the University of Manitoba Human Research Ethics Board (H2015:137) and Research Impact Committee (R12015:048). Measures were completed by every client on a session-to-session basis, immediately after each session, as part of routine monitoring of clinical progress. ‘Sessions’ included the initial intake assessment at the clinic, each CBTm class, and each small group CBT session following the classes (if applicable). The intake assessment served as the baseline and each subsequent class attended in chronological order, regardless of intervention cycle, served as an individual time-point (up to a maximum of 4). The measures completed immediately before the first small group CBT session served as the study’s follow-up. Classes were held weekly, with only slight variation due to clinician availability or holidays. The mean duration between the intake assessment and the first attended CBTm class was 4 weeks and the median was 2 weeks. The mean duration between the last attended class and follow-up was 12 weeks and the median was 9 weeks.

### Measures

#### Sociodemographic factors and dropout

Sociodemographic factors of interest (age, sex, marital status, education and employment) were obtained from a self-report questionnaire found in clients’ medical charts. Clients completed this questionnaire on the day of their intake assessment at the clinic. The primary mental health diagnosis was also obtained from clients’ medical charts, either through the CBTm class assessment form or other relevant notes and assessments completed at the intake assessment. Dropout data was obtained using class attendance forms completed at every class.

#### Acceptability and baseline predictors of class completion

Clients’ self-reported acceptability of the CBTm classes was assessed using two items from the evaluation form they completed immediately after each session. The first item asks the respondent how useful they find the session and is rated on a 5-point Likert scale ranging between “1=Not very useful” and “5 = Extremely useful”. The second item is a dichotomous Yes/No question asking the respondent if they would attend another session like the one they just attended. These two items were chosen as they are easy to report and are completed by most clients. Remaining items on the evaluation form were not chosen for this study as they were often left blank by clients; these three qualitative items ask the respondent to [1] list three things they learned [2] describe what they like about the session and [3] describe what could be improved. Future studies may wish to use formal measures of satisfaction as these were not implemented in the current study. Generalized Anxiety Disorder 7-item (GAD-7) score, Patient Health Questionnaire 9-item (PHQ-9) score, sex, education, and mental health diagnoses at the time of intake assessment were the baseline variables of interest in predicting completion of 4 or more classes.

#### Anxiety and depressive symptoms

The Generalized Anxiety Disorder 7-item (GAD-7) scale [33] and the Patient Health Questionnaire 9-item (PHQ-9) scale [34] were used to assess changes in anxiety and depressive symptoms, respectively. Both scales ask the respondent to reflect on how often they have been bothered by their symptoms over the past 2 weeks and rate items on a 4-point Likert scale ranging between 0=“Not at all” and 3=“Nearly every day”. A large body of literature shows the GAD-7 and PHQ-9 have good test-retest reliability and validity in measuring symptom severity in the general population [33–36]. These scales are also useful in monitoring symptom change across time [33, 35, 37].

We used two separate methods to assess whether clients experienced clinically significant improvements in symptoms with the CBTm intervention. The first was based on McMillan et al.’s recommendations [38]. Their definition of clinically significant change comprised of three components; applied to anxiety they are: (a) mean GAD-7 score at follow-up < 8 [33] (b) improvement greater than, or equal to, the minimum clinically important difference (MCID), and (c) at least medium effect sizes (≥ 0.50). Applied to depression they are: (a) mean PHQ-9 score at follow-up < 10 [34] (b) improvement greater than, or equal to, the MCID, and (c) at least medium effect sizes (≥ 0.50). The MCID noted above is the smallest difference in score considered to be clinically important, and was determined to be 5 in our study for both the GAD-7 and PHQ-9 using Lowe et al.’s methodology [37]. According to this methodology, the MCID for a measure can be estimated using the following calculation (*r* = reliability coefficient):

$$ MCID=1.96\times \left({SD}_{baseline}\times \sqrt{1-r}\right) $$

Another method to calculate clinically significant change was applied to the GAD-7 and PHQ-9 in our study [39]. According to this methodology, one can compare the mean score and standard deviation of a clinical population with that of a referential “normal” population to determine the extent to which change after treatment is clinically meaningful. We compared the scores from our sample to healthy samples from other studies [33, 34]. The calculation is:

$$ \frac{\left({Mean}_{clin}\times {SD}_{norm}\right)+\left({Mean}_{norm}\times {SD}_{clin}\right)}{SD_{norm}+{SD}_{clin}} $$

Using this calculation, one would expect clinically significant change if the GAD-7 score change is ≥6.52 for anxiety, and if the PHQ-9 score change is ≥7.60 for depression.

### Analytic strategy

Statistical analysis was performed using SPSS (version 24) and STATA (version 13.1). Adjusted odds ratios were calculated using a logistic regression model to assess baseline predictors of class completion. The variables of interest were regressed against a binary variable indicating completion of at least 4 classes. Descriptive statistics were also obtained for the sample.

The independent variable was the number of CBTm classes attended by a subject and the dependent variable was the score on the GAD-7 and PHQ-9. Primary analysis used a mixed-effects linear regression model to estimate the effect of the number of classes on the outcome measures. The model allowed the data to be analyzed as a within-subject design to examine the change in scores across repeated outcome measures in the same subject (up to six time-points were clustered within individual). The individual level was the only level in the model as clients could not be clustered by groups or treatment setting given the data available. Maximum likelihood estimation was used in this model. The fixed effects were the number of classes (a continuous variable), the effect of the gap between baseline and the first attended class (a binary dummy variable coded 1 after the gap has occurred), and the effect of the gap between the last attended class and follow-up (a binary dummy variable coded 1 starting with follow-up) on GAD-7 and PHQ-9 scores. The random effects examine the difference between individuals with respect to their baseline score and the changes over time. Other variables (sex, education, and mental health diagnoses) were not included or adjusted for in this analysis as they did not vary within individuals. Effect sizes (Cohen’s *d*) of the regression model estimates were also obtained using the baseline standard deviation.

Secondary analysis calculated the mean differences in outcome measure scores between baseline and follow-up. Percent reductions in symptoms were calculated using the mean difference in outcome measure scores as the dividend and the mean baseline score as the divisor. Effect sizes (Cohen’s *d*) of the mean differences were also obtained.

## Results

### Sociodemographic factors and dropout

Of the 655 clients assessed for eligibility for the CBTm classes from November 2014 to December 2016, 533 attended at least one class (10 of these clients did not fill out any measures and were not included in analysis). The 122 clients who did not attend any class either chose not to for personal reasons or met exclusion criteria. Limited dropout information precludes us from providing further detail. Among those who attended, 333 (62.5%) completed 4 or more classes and 200 (37.5%) dropped out. We define dropout as completing less than 4 classes, regardless of reason for dropout. The mean and median number of classes attended by this sample were 3.5 (SD = 1.7) and 4 (IQR = 2 to 4), respectively. 179 clients (53.8% of those eligible) moved on to receive small group CBT. The mean age of the analyzed sample was 39.3 (SD = 13.8), and more than half were female (58.9%). There were a broad range of primary mental health diagnoses which are reported in Table 1.

**Table 1:** Sociodemographic Characteristics at Intake Assessment (*n* = 523)

Variable	
Age	
Years, mean (SD)	39.3 (13.8)
Sex, *n* (%)	
Male	178 (34.0)
Female	308 (58.9)
Unknown^a^	37 (7.1)
Marital Status, *n* (%)	
Married or common law	180 (34.4)
Separated, divorced, or widowed	58 (11.1)
Never married	237 (45.3)
Unknown^a^	48 (9.2)
Education, *n* (%)	
No high school graduation	56 (10.7)
High school graduation	117 (22.4)
Some postsecondary	107 (20.5)
Trade, college, or university certificate or diploma	47 (9.0)
University Degree	116 (22.1)
Unknown^a^	80 (15.3)
Employment, *n* (%)	
Paid employment or retired	251 (48.0)
Unemployed	160 (30.6)
Student	28 (5.4)
Unknown^a^	84 (16.1)
Primary mental health diagnosis, *n* (%)	
Major Depressive Disorder	137 (26.2)
Generalized Anxiety Disorder	101 (19.3)
Social Anxiety Disorder	43 (8.2)
Obsessive Compulsive Disorder	34 (6.5)
Persistent Depressive Disorder	32 (6.1)
Posttraumatic Stress Disorder	31 (5.9)
Panic Disorder	24 (4.6)
Bipolar Disorder	24 (4.6)
Anxiety Not Otherwise Specified	19 (3.6)
Depression Not Otherwise Specified	18 (3.4)
Other^b^	60 (11.5)

^a^Respondent failed to complete item. ^b^Includes specific phobia, post schizophrenic depression, postpartum depression, alcohol and substance use disorders, eating disorders, personality disorders, somatic symptom disorders, neurodevelopmental disorders, cognitive disorders

### Acceptability and baseline predictors of class completion

Mean scores on the usefulness item ranged between 3.9 and 4.1 (SD range = 0.80 to 0.89). The proportion of clients who indicated they would attend another session was consistently over 90%, with a range of 94 to 99%, depending on the session. Two significant baseline predictors of CBTm class completion were found. First, clients with a higher baseline score on the PHQ-9 were significantly less likely to complete 4 classes (OR = 0.95 [95%CI 0.91 to 0.99], *p* < 0.05). Second, clients who did not complete high school (did not graduate and/or receive diploma) were also significantly less likely to complete 4 classes (OR = 0.39 [95%CI 0.20 to 0.75], *p* < 0.05). All other baseline variables did not significantly predict class completion: GAD-7 score (*p* = 0.93), sex (*p* = 0.92), mental health diagnosis (*p* = 0.37).

### Anxiety symptoms

The mean baseline score for anxiety symptom severity was GAD-7 = 12.6 (SD = 5.8). The mixed-effects linear regression indicated a statistically significant decline in symptoms of anxiety when attending CBTm classes (mean GAD-7 score change per class = − 0.52 [95%CI, − 0.74 to − 0.30], *p* < 0.001). There was significant variation in effect across individuals based on the regression model. The Cohen’s effect size for this analysis was *d* = 0.36, a small effect [40]. The regression model controlled for the significant decline in symptoms between baseline and the first class, and the non-significant increase in symptoms between class 4 and follow-up (Table 2).

**Table 2:** Mean Changes in Outcome Measure Scores Using Mixed-Effects Linear Regression

Outcome Measure	Phase of Therapy	Mean Change^d^ (95% CI)
GAD-7	Intake Assessment^a^	− 0.98** (− 1.58 to − 0.38)
	CBTm Classes^b^	−0.52** (− 0.74 to − 0.30)
	Treatment Gap^c^	0.79 (− 0.13 to 1.71)
PHQ-9	Intake Assessment^†^	−0.82* (− 1.51 to − 0.13)
	CBTm Classes^b^	− 0.65** (− 0.89 to − 0.40)
	Treatment Gap^§^	1.40* (0.43 to 2.36)

^a^Time between intake assessment (baseline) and CBTm Class 1. ^b^Effect of CBTm classes alone. Controlled for time between baseline and CBTm class 1 and treatment gap between CBTm class 4 and first small group CBT session (follow-up). ^c^Reflects time between CBTm class 4 and follow-up. ^d^For CBTm classes, reflects mean change per CBTm class attended

*CBTm* Cognitive Behaviour Therapy with Mindfulness, *GAD-7* Generalized Anxiety Disorder 7-item scale, *PHQ-9* Patient Health Questionnaire 9-item scale

**P ≤ 0.001. *P ≤ 0.05

The mean change in the GAD-7 score between baseline and follow-up was − 2.40 (95%CI, − 3.38 to − 1.41) – an 18% (95%CI, 9–24%) reduction in anxiety symptoms (Table 3). The Cohen’s effect size for this analysis was *d* = 0.41, a small effect. These results were not clinically significant according to Mcmillan et al.’s definition [38]. Specifically, we calculated (a) the post-treatment GAD-7 score of 10.2 was greater than the cutoff of 8 (b) the improvement of 2.40 did not meet the MCID of 5 which was calculated and (c) the effect size of 0.41 was less than the threshold of 0.50. These findings were also not clinically significant according to Evans et al.’s definition [39]. Specifically, the GAD-7 score improvement of 2.40 did not meet the calculated value of 6.52.

**Table 3:** Mean Changes and Percent Reduction in Outcome Measure Scores between Baseline and Follow-up^a^

Outcome Measure	Mean Change (95% CI)	Percent Reduction, % (95% CI)
GAD-7	−2.40 (−3.38 to − 1.41)	18 (9 to 24)
PHQ-9	−1.98 (− 3.13 to − 0.83)	13 (3 to 18)

^a^Intake assessment served as study baseline. First small group CBT session served as study follow-up

*GAD-7* Generalized Anxiety Disorder 7-item scale, *PHQ-9* Patient Health Questionnaire 9-item scale

### Depressive symptoms

The mean baseline score for depression symptom severity was PHQ-9 = 15.2 (SD = 6.8). The mixed-effects linear regression indicated a statistically significant decline in symptoms of depression when attending CBTm classes (mean PHQ-9 score change per class = − 0.65 [95%CI, − 0.89 to − 0.40], *p* < 0.001). There was significant variation in effect across individuals based on the regression model. The Cohen’s effect size for this analysis was *d* = 0.38, a small effect [40]. The regression model controlled for the significant decline in symptoms between baseline and the first class, and the significant increase in symptoms between class 4 and follow-up (Table 2).

The mean change in the PHQ-9 score between baseline and follow-up was − 1.98 (95%CI, − 3.13 to − 0.83) – a 13% (95%CI, 3–18%) reduction in depressive symptoms (Table 3). The Cohen’s effect size for this analysis was *d* = 0.29, a small effect. These results were not clinically significant according to Mcmillan et al.’s definition [38]. Specifically, we calculated (a) the post-treatment PHQ-9 score of 13.2 was greater than the cutoff of 10 (b) the improvement of 1.98 did not meet the MCID of 5 which was calculated and (c) the effect size of 0.29 was less than the threshold of 0.50. These findings were also not clinically significant according to Evans et al.’s definition [39]. Specifically, the PHQ-9 score improvement of 1.98 did not meet the calculated value of 7.60.

## Discussion

The current study demonstrates that a 4-session, large, Cognitive Behaviour Therapy with Mindfulness (CBTm) class intervention is acceptable and may be associated with an improvement in anxiety and depressive symptoms. To our knowledge, this is the first study to investigate a brief, low intensity, large group CBT intervention which incorporates mindfulness. Our findings expand upon previous work from our group which found large group CBT classes were useful, led to modest improvements in anxiety symptoms, and reduced wait times for conventional CBT group therapy [20].

Our first hypothesis that the CBTm classes would be acceptable, both in terms of client feedback and retention rates, was supported by the results. Clients reported they found the classes useful and a significant proportion (over 90%) indicated they would attend another session. These findings remained stable through the 4 sessions, suggesting the classes are a viable strategy to facilitate CBT access and to engage and maintain the interest of a large number of clients. Successful engagement is a key advantage of this service delivery model as traditional CBT delivery often fails to provide clients with equitable and timely access to CBT skills at their time of most need. The CBTm classes had a dropout rate of 37.5%, which is consistent with similar large group CBT interventions found in the literature [26–28]. More than half (53.8%) of eligible clients (those that completed the 4-session intervention) moved on to conventional small group CBT therapy. Future research should investigate long-term dropout rates and specific reasons for dropout as these were not assessed in this study.

Our second hypothesis that clients would experience improvements in anxiety and depressive symptoms was supported by the results, although effects were small. Primary analysis showed statistically significant improvement in both anxiety and depressive symptoms, with small effect sizes (*d* = 0.36 and *d* = 0.38 for anxiety and depressive symptoms, respectively). Our effect sizes are smaller than those reported in recent UK studies investigating large-group CBT [26, 28]. The first study was a randomized controlled trial showing a one-day large CBT workshop had a medium effect in reducing depressive (*d* = 0.55) and anxiety symptoms (effect size not reported) 12 weeks post intervention [26]. The other study investigated a 6-session large-group CBT intervention across 5 services over time. Investigators reported similar values in improving short-term depressive (*d* = 0.59) and anxiety symptoms (*d* = 0.70) [28]. Our smaller effect sizes make it unclear if clients attending the CBTm classes achieved clinically significant improvements in symptoms.

To further elucidate whether clients’ symptom improvement was clinically significant, we applied two separate definitions of ‘clinically significant change’ from the literature [38, 39]. This analysis again demonstrates clients did not experience clinically meaningful change in either anxiety or depressive symptoms. These findings may be partially explained by the length of the CBTm intervention as four 90 min sessions is brief when compared to formal CBT programs [10] and Delgadillo et al.’s study [28]. Dosing of psychotherapy is an important factor which influences efficacy, thus the brevity of our intervention makes the smaller effect sizes understandable. However, Horrell et al.’s trial [26] had higher effect sizes than CBTm, despite being similar in length, which suggests other factors may better explain our study’s lack of clinical significance. One possible explanation is our sample included a broad range of mental health diagnoses, whereas the extant literature limited their sample to those with depression [26] or a mix of anxiety and depression [28]. It is also possible the CBTm intervention is simply less effective than these existing interventions at treating anxiety and depression.

The CBTm intervention’s lower effectiveness in treating anxiety and depression may be partially explained by the fact that our sample had greater baseline symptom severity compared to extant literature [26, 28]. Recent work demonstrates greater symptom severity is associated with dropout [41] and poorer clinical outcomes [42, 43] in psychotherapy. Consistent with this, one service in Delgadillo et al.’s study [28] with greater symptom severity attained a lower effect size (*d* = 0.48) for anxiety relative to the other services. In our study, clients with more severe depression or lower education were more likely to dropout, which may also have impacted the intervention’s effectiveness. One possible explanation for the dropout, initially speculated by Fernandez et al. [41], is that the major symptomology of depression (namely diminished interest, poor concentration, lower energy, hopelessness, social withdrawal) may make it more difficult to engage and maintain the interest of this population. This would seem especially relevant in a large-group setting where clients require significant motivation and attention to both attend class and to adequately absorb information delivered in a didactic fashion. Interestingly, there was a significant increase in depressive symptoms in the treatment gap between the last attended CBTm class and follow-up. This demonstrates the importance of continual engagement and treatment for this population as our results seem to suggest they are prone to relapse after disengaging from the CBTm intervention for some time. In regards to lower education and dropout, it is well known these two variables are associated in psychotherapy [44], but it is less clear as to why. It is possible lack of insight into their mental illness, differences in expectations of therapy, or difficulty understanding class content played a role. What is clear is future practitioners delivering low intensity CBT should implement strategies to more effectively engage and treat those clients with more severe depression or lower education. Perhaps small group or individual psychotherapy, with more personalized content for specific symptom or education levels, is an appropriate approach to treatment in these vulnerable populations.

Study limitations include the lack of an appropriate control group to serve as a comparison to the CBTm class intervention. As Delgadillo and colleagues discussed, it is possible symptom improvement was observed due to natural variation resulting from the passage of time, general contact with healthcare professionals, or contact with other clients [28]. Investigating the effects of large group CBT in a natural tertiary care setting is a strength as it offers an accurate estimate of the “real world” effectiveness of this intervention. This, coupled with a large sample size (*n* = 523), suggests our findings likely have strong external validity. Future studies should implement a more rigorous randomized and controlled study design to clarify the effects of the CBTm intervention and minimize potential confounders. It may also be advantageous to benchmark outcomes from the CBTm intervention with other interventions at our service, such as conventional small group CBT. Having this comparison data available would strengthen the conclusions drawn about the relative effectiveness of CBTm. Other limitations include the indeterminate reliability of using single item measures to assess acceptability of the CBTm intervention, as well as not using a standardized diagnostic tool to establish a mental health diagnosis at screening. Another notable limitation is we allowed clients to access meditations at their own discretion – this complicates assessment of treatment adherence and carries the risk of adverse effects as there are meditations of uncertain quality available online. Moving forward it may be helpful to encourage clients to limit their use to a few high quality meditations, which we provide as resources.

## Conclusions

This study offers preliminary evidence supporting the acceptability and potential applicability of large group psychoeducational CBT classes in Canada. Our results demonstrate CBTm classes are a viable strategy to facilitate access and to engage and maintain clients’ interest in pursuing CBT. The classes also have a modest yet statistically significant effect in treating symptoms of anxiety and depression. This study meets the national goal of providing “clinical outcomes data from a real-world setting” for CBT [15], but further research needs to be done to address our study’s limitations and refine our findings. As additional evidence confirms the effectiveness of these classes in Canada, they may become a key component in a client’s healthcare journey as they provide psychoeducation and strategies to manage symptoms at a time when they may otherwise have difficulty accessing therapy.

## Abbreviations

CBT: Cognitive Behaviour Therapy
CBTm: Cognitive Behaviour Therapy with Mindfulness
DMN: Default mode network
GAD-7: Generalized Anxiety Disorder 7-item Scale
MBCT: Mindfulness-based cognitive therapy
MBSR: Mindfulness-based stress reduction
MCID: Minimum clinically important difference
PHQ-9: Patient Health Questionnaire 9-item Scale

## References

[1] Mcrae L, Donnell SO, Loukine L, Rancourt N, Pelletier C (2016). Mood and anxiety disorders in Canada, 2016. Heal Promot Chronic Dis Prev Canada.

[2] Katzman MA, Bleau P, Blier P, Chokka P, Kjernisted K, Van AM (2014). Canadian clinical practice guidelines for the management of anxiety, posttraumatic stress and obsessive-compulsive disorders. BMC Psychiatry.

[3] Aquin JP, El-gabalawy R, Sala T, Sareen J (2017). Anxiety disorders and general medical conditions: current research and future directions. Focus (Madison).

[4] Gagnon LM, Patten SB (2002). Major depression and its association with long-term medical conditions. Can J Psychiatr.

[5] Essau Cecilia A., Lewinsohn Peter M., Olaya Beatriz, Seeley John R. (2014). Anxiety disorders in adolescents and psychosocial outcomes at age 30. Journal of Affective Disorders.

[6] Kessler RC, Berglund P, Demler O, Jin R, Koretz D, Merikangas KR (2003). The epidemiology of major depressive disorder. JAMA..

[7] Plaisier I., Beekman A.T.F., de Graaf R., Smit J.H., van Dyck R., Penninx B.W.J.H. (2010). Work functioning in persons with depressive and anxiety disorders: The role of specific psychopathological characteristics. Journal of Affective Disorders.

[8] Stewart WF, Ricci JA, Chee E, Hahn SR, Morganstein D (2003). Cost of lost productive work time among US Workers with depression. J Am Med Assoc.

[9] Wittchen H-U (2002). Generalized anxiety disorder: prevalence, burden, and cost to society. Depress Anxiety.

[10] Parikh Sagar V., Quilty Lena C., Ravitz Paula, Rosenbluth Michael, Pavlova Barbara, Grigoriadis Sophie, Velyvis Vytas, Kennedy Sidney H., Lam Raymond W., MacQueen Glenda M., Milev Roumen V., Ravindran Arun V., Uher Rudolf (2016). Canadian Network for Mood and Anxiety Treatments (CANMAT) 2016 Clinical Guidelines for the Management of Adults with Major Depressive Disorder. The Canadian Journal of Psychiatry.

[11] Cuijpers P, Berking M, Andersson G, Quigley L, Kleiboer A, Dobson KS (2013). A meta-analysis of cognitive-Behavioural therapy for adult depression, alone and in comparison with other treatments. Can J Psychiatr.

[12] Butler AC, Chapman JE, Forman EM, Beck AT (2006). The empirical status of cognitive-behavioral therapy: a review of meta-analyses. Clin Psychol Rev.

[13] Hofmann SG, Smits J a J (2008). Cognitive-behavioral therapy for adult anxiety disorders: a meta-analysis of randomized placebo-controlled trials. J Clin Psychiatry.

[14] Beck JS (2011). Cognitive behavior therapy: basics and beyond.

[15] Payne KA, Myhr G (2010). Increasing access to cognitive-Behavioural therapy (CBT) for the treatment of mental illness in Canada: a research framework and call for action. Healthc Policy.

[16] Mojtabai Ramin (2009). Unmet Need for Treatment of Major Depression in the United States. Psychiatric Services.

[17] Kohn R, Saxena S, Levav I, Saraceno B (2004). The treatment gap in mental health care. Bull World Health Organ.

[18] Parikh SV (2015). Improving access to psychosocial treatments— integrating patient, provider, and systems approaches. Can J Psychiatr.

[19] Delgadillo Jaime, McMillan Dean, Lucock Mike, Leach Chris, Ali Shehzad, Gilbody Simon (2013). Early changes, attrition, and dose-response in low intensity psychological interventions. British Journal of Clinical Psychology.

[20] Palay Joshua, Wong Jacquelyne Y, Randall Jason R, Sala Tanya, Bolton James M, Furer Patricia, Bolton Shay-Lee, Whitney Debbie L, Thakur Vishal, Parikh Sagar V, Sareen Jitender (2018). Feasibility of large group cognitive behavioural therapy education classes for anxiety disorders. European Journal for Person Centered Healthcare.

[21] Kabat-Zinn J (2003). Mindfulness-based interventions in context: past, present, and future. Clin Psychol Sci Pract.

[22] Hofmann SG, Sawyer AT, Witt AA, Oh D (2010). The effect of mindfulness-based therapy on anxiety and depression: a meta-analytic review. J Consult Clin Psychol.

[23] Hofmann SG, Gomez AF (2017). Mindfulness-based interventions for anxiety and depression. Psychiatr Clin North Am.

[24] Raffone Antonino, Marzetti Laura, Del Gratta Cosimo, Perrucci Mauro Gianni, Romani Gian Luca, Pizzella Vittorio (2019). Toward a brain theory of meditation. Progress in Brain Research.

[25] Sipe WEB, Eisendrath SJ (2012). Mindfulness-based cognitive therapy: theory and practice. Can J Psychiatr.

[26] Horrell L, Tylee T, Schmidt UH, Murphy CL, Goldsmith KA, Bonin E, et al. One-day cognitive–behavioural therapy self-confidence workshops for people with depression: randomised controlled trial. Br J Psychiatry. 2014;204:222–33. 10.1192/bjp.bp.112.121855 24357574

[27] Burns P, Kellett S, Donohoe G (2016). “Stress control” as a large group psychoeducational intervention at step 2 of IAPT services: acceptability of the approach and moderators of effectiveness. Behav Cogn Psychother.

[28] Delgadillo Jaime, Kellett Stephen, Ali Shehzad, McMillan Dean, Barkham Michael, Saxon David, Donohoe Gill, Stonebank Heather, Mullaney Sarah, Eschoe Patricia, Thwaites Richard, Lucock Mike (2016). A multi-service practice research network study of large group psychoeducational cognitive behavioural therapy. Behaviour Research and Therapy.

[29] Kabat-Zinn J (1982). An outpatient program in behavioral medicine for chronic pain patients based on the practice of mindfulness meditation: results. Gen Hosp Psychiatry.

[30] Evans S, Ferrando S, Findler M, Stowell C, Smart C, Haglin D (2008). Mindfulness-based cognitive therapy for generalized anxiety disorder. J Anxiety Disord.

[31] Segal Z, Williams JMG, Teasdale JD (2002). Mindfulness-based cognitive therapy for depression: a new approach to preventing relapse.

[32] Chiesa Alberto, Serretti Alessandro (2009). Mindfulness-Based Stress Reduction for Stress Management in Healthy People: A Review and Meta-Analysis. The Journal of Alternative and Complementary Medicine.

[33] Spitzer RL, Kroenke K, Williams JBW, Lowe B (2006). A brief measure for assessing generalized anxiety disorder. Arch Intern Med.

[34] Kroenke K, Spitzer RL, Williams JBW (2001). The PHQ-9: validity of a brief depression severity measure. J Gen Intern Med.

[35] Lowe B, Decker O, Muller S, Brahler E, Schellberg D, Herzog W (2008). Validation and standardization of the generalized anxiety disorder screener (GAD-7) in the general population. Med Care.

[36] Martin A, Rief W, Klaiberg A, Braehler E (2006). Validity of the brief patient health questionnaire mood scale (PHQ-9) in the general population. Gen Hosp Psychiatry.

[37] Lowe B, Unutzer J, Callahan CM, Perkins AJ, Kroenke K (2004). Monitoring depression treatment outcomes with the patient health Questionnaire-9. Med Care.

[38] McMillan Dean, Gilbody Simon, Richards David (2010). Defining successful treatment outcome in depression using the PHQ-9: A comparison of methods. Journal of Affective Disorders.

[39] Evans C, Margison F, Barkham M (1998). The contribution of reliable and clinically significant change methods to evidence-based mental health. Evid Based Ment Heal.

[40] Sawilowsky SS (2009). New effect size rules of thumb. J Mod Appl Stat Methods.

[41] Fernandez Ephrem, Salem Dara, Swift Joshua K., Ramtahal Nirvana (2015). Meta-analysis of dropout from cognitive behavioral therapy: Magnitude, timing, and moderators. Journal of Consulting and Clinical Psychology.

[42] Delgadillo J, Asaria M, Ali S, Gilbody S (2016). On poverty, politics and psychology: the socioeconomic gradient of mental healthcare utilisation and outcomes. Br J Psychiatry.

[43] Firth Nick, Barkham Michael, Kellett Stephen, Saxon Dave (2015). Therapist effects and moderators of effectiveness and efficiency in psychological wellbeing practitioners: A multilevel modelling analysis. Behaviour Research and Therapy.

[44] Wierzbicki M, Pekarik G (1993). A meta-analysis of psychotherapy dropout. Professional psychology: research and practice.

